# Kinetics of Zinc Evaporation from Aluminium Alloys Melted Using VIM and ISM Technologies

**DOI:** 10.3390/ma14216641

**Published:** 2021-11-04

**Authors:** Albert Smalcerz, Bartosz Wecki, Leszek Blacha, Jerzy Labaj, Maciej Jodkowski, Adrian Smagor

**Affiliations:** 1 Department of Industrial Informatics, Faculty of Materials Science, Silesian University of Technology, Krasinskiego 8, 40-019 Katowice, Poland; adrian.smagor@polsl.pl; 2 Department of Testing and Certification “ZETOM”, Ks. Herberta Bednorza 17, 40-384 Katowice, Poland; bartosz.wecki@zetom.eu (B.W.); maciej.jodkowski@zetom.eu (M.J.); 3 Department of Metallurgy and Recycling, Faculty of Materials Science, Silesian University of Technology, Krasinskiego 8, 40-019 Katowice, Poland; leszek.blacha@polsl.pl (L.B.); jerzy.labaj@polsl.pl (J.L.)

**Keywords:** vacuum refining of metals, vacuum induction melting (VIM), induction skull melting (ISM)

## Abstract

Using a vacuum during the smelting and refining of alloys removes dissolved gasses, as well as impurities with high vapour pressure. When smelting is carried out in vacuum induction furnaces, the intensification of the discussed processes is achieved by intensive mixing of the bath, as well as an enhanced mass exchange surface (liquid metal surface) due to the formation of a meniscus. This is due to the electromagnetic field applied to the liquid metal. This study reports the removal of zinc from the Al-Zn alloy containing 6.3 wt.%. Zn. The experiments were carried out with the use of two types of metallurgical devices: the VIM and ISM furnaces. For the experiments carried out in the crucible induction furnace, reduction in the operating pressure in the furnace from 1000 Pa to 10 Pa, together with the increase in temperature from 953 K to 1103 K, is accompanied by a decrease in zinc content in the alloy from 6 to 96%, compared with the initial value. Simultaneously, the overall mass transfer coefficient *k_Zn_* increases from 5.15 × 10^−6^ to −1.49 × 10^−4^ ms^−1^. For the experiments carried out in the furnace with a cold crucible (*T* = 953 K), a reduction in the operating pressure in the furnace from 1000 Pa to 10 Pa resulted in a decrease in zinc content in the alloy from 18 to 80%, compared with the initial value. For comparison, the experiments carried out in the crucible induction furnace at 953 K showed a reduction in zinc content in the alloy from 6 to 50%, which means that more intense zinc evaporation is seen in the furnace with a cold crucible. Comparison of ISM and VIM technologies in the removal efficiency of the Al-Zn alloy indicates a higher removal efficiency using the first technology, which, using the same conditions, achieves 80% of the removal efficiency of the component.

## 1. Introduction

The dynamic development of the world economy requires the supply of more and more materials that meet high-quality requirements. Metals belong to the group of strategic construction materials, but the basic issue of their use in advanced solutions is their purity. Physicochemical properties of metals, such as chemical affinity for oxygen, reductions, surface tension, viscosity, and gas vapour pressure, constitute basic barriers that, in the currently used refining technologies, are limitations for the level of removal of impurities in metals and their alloys. The emerging solutions based on scientific studies and the increasing technical possibilities of building advanced metallurgical structures create new possibilities, especially in the area of selective metal preparation and purification.

In the literature on the subject, one can find a well-documented database of results concerning metal refining processes with the use of melting aggregates based on inductive remelting under reduced pressure conditions, such as VIM. However, the emerging base of secondary raw materials and the exhausting existing resources of natural resources mean that the search for solutions that have the potential in the area of processing and selective recovery of materials with complex chemical compositions are particularly of interest. Such solutions include the induction skull melting (ISM) technology, which is based on induction melting using a phenomenon similar to levitation so that the melted metal does not come into contact with the crucible material, which can also often introduce contamination to the metal.

Due to the very high intensity of the interaction of electromagnetic forces, the liquid metal is affected by large forces that significantly increase the interface between the liquid and gas phases. Taking into account the kinetic analysis of metal refining processes, a considerable role in this process is played by the mass transfer surface, which is the interface.

The research results presented in this article constitute an innovative approach to the refining of metals, including zinc and aluminium. Thus far, due to high costs, ISM remelting solutions are used in the technologies of obtaining titanium, while the use of non-ferrous metals in this technology requires a number of tests to supplement the knowledge in this field. Comparison of ISM and VIM technologies in the removal efficiency of the Al-Zn alloy indicates a higher removal efficiency using the first technology, which, using the same conditions, achieves 80% of the removal efficiency of the component.

The phenomenon of evaporation of volatile metal bath components can be observed in numerous processes of both extractive metallurgy and refining. In some of them, this phenomenon can be considered positive, while in others, however, to the contrary. In order to intensify the evaporation process or to restrain it, one must necessarily know the factors determining its rate. Among the most important of these factors are the pressure inside the system, the type of gas atmosphere, the temperature, and the hydrodynamic properties of the system in which the metallurgical process is carried out. Crucible-based vacuum induction furnaces (VIM technology), such as cold crucible induction furnaces (ISM technology), are melting machines used in modern refining or smelting processes. In these operations, due to the use of vacuum, it is possible to evaporate its components, characterised by high vapour pressure from the metallic bath.

The source of the electromagnetic field in induction furnaces is a coil made of copper. Taking into account the construction of crucible, we have two kinds of induction furnaces:

- Induction furnace with a non-conductive crucible, e.g., ceramic crucible;
- Induction furnace with a conductive crucible, e.g., graphite crucible or copper crucible in case of the cold crucible furnace.

**Figure 1:**
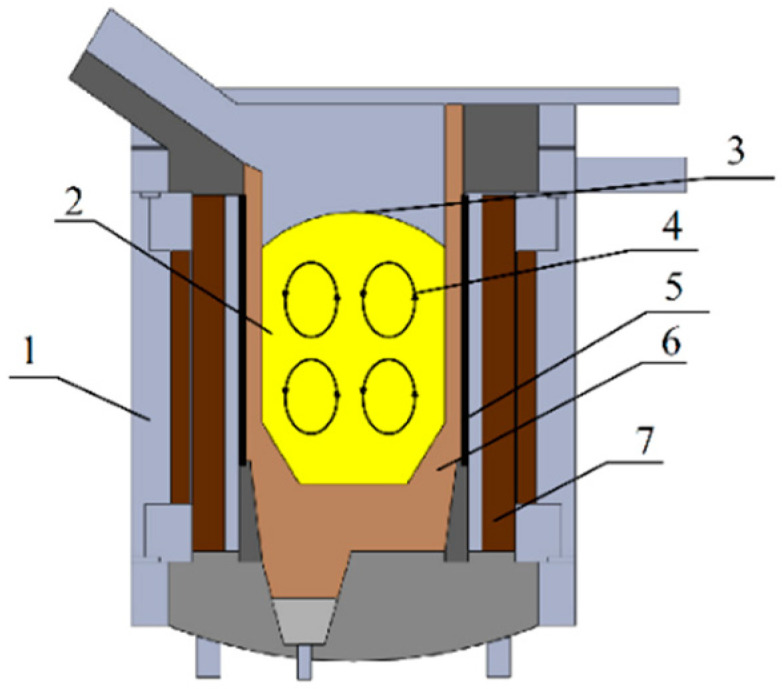
Scheme of the induction crucible furnace: 1—steel construction; 2—liquid metal; 3—meniscus; 4—mixing of metal; 5—inductor; 6—crucible; 7—ferromagnetic core [1].

Magnetic cores or magnetic field concentrators are applied to concentrate the electromagnetic field. Figure 1 shows a furnace in which the magnetic core is longer than the induction coil.

The electromagnetic field generated by the alternating electric current flowing through the inductor, directly or indirectly, affects the workpiece placed inside the crucible. Eddy currents induced in the workpiece are responsible for heat generation in the metal. As a result of the interaction, the Lorentz force is generated [2,3,4,5]. It causes a convex meniscus of free surface and intensive stirring of the metal (Figure 1).

**Figure 2:**
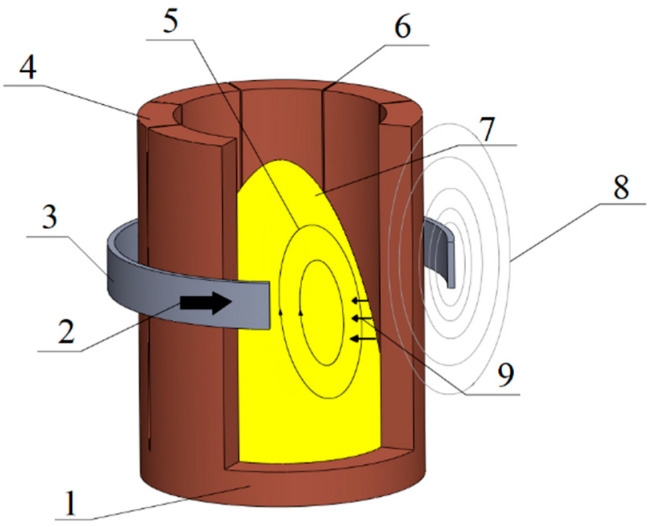
Scheme of the induction furnace with a cold crucible: 1—crucible; 2—current; 3—inductor; 4—segment; 5—melt flow; 6—slit; 7—meniscus; 8—magnetic field; 9—force density [1].

The application of direct heating in the crucible may cause intense reactions of the crucible with the metal, e.g., titanium or zircon [6]. It causes increased interest in indirect heating in cold crucible furnaces (Figure 2).

Another technology for induction smelting, one that partially eliminates the disadvantages of classical crucible smelting, is smelting in induction furnaces with a so-called cold crucible (Figure 2).

The same phenomena occur in cold crucible furnaces (ISM technology). The water-cooled crucible is mostly made of copper [7,8,9,10,11]. However, this means a limited transfer of energy to the workpiece. Eddy currents induced on the inner wall of the crucible are the source of the secondary electromagnetic field induced in the workpiece [12,13].

When metals or their alloys are melted in induction furnaces (VIM or ISM), the surface area of the metal is strongly dependent on the electromagnetic field acting on the alloy and the properties of the liquid metal. This is evinced by the formation of a distinct meniscus on the surface of the bathtub. Increasing the surface of the liquid alloy can significantly intensify the discussed evaporation process. 

The aims of this work include the following:

- Approximating the surface of liquid Al-Zn alloy melted using VIM and ISM technologies;
- Laboratory smelting of these alloys in variable pressure and temperature;
- Estimation of the overall mass transfer coefficient of zinc on the basis of experimental data;
- Determination of the stages in finding the velocity of the analysed evaporation process.

The selection of the alloy Al-Zn which the tests were conducted resulted from the fact that zinc as a component of this alloy is characterised by much higher vapour pressure, compared with aluminium. The paper presents the kinetic analysis of zinc evaporation from the Al-Zn alloy containing 6.3 wt.%. Zn. The experiments were carried out with the use of two types of metallurgical devices—the VIM and ISM furnaces, ranging from 953 K to 1103 K and a wide range of operating pressures, from 10 Pa to 1000 Pa.

## 2. Materials and Methods

### 2.1. A Thermodynamic Condition of Zinc Evaporation from Liquid Aluminium

The potential for evaporation of the A element from a two-component A-B alloy is determined by the evaporation coefficient Ω which is described by the equation [14].

(1) $$\Omega =\frac{\gamma_{A}\cdot P_{A}^{0}}{\gamma_{B}\cdot P_{B}^{0}},$$

where *P_A_*^0^, *P_B_*^0^—equilibrium vapour pressures over pure components *A* and *B*, respectively; *γ_A_*, *γ_B_*—coefficients of activity in the alloy for *A* and *B*, respectively.

For the two-component *A-B* alloy, with Ω ≈ 1, it is assumed that the chemical composition of the alloy does not change during the smelting process. When the coefficient value is Ω > 1, we can infer a loss (evaporation) of the *A* component from the alloy relative to the *B* component. When the following condition is met: Ω < 1, we can infer a loss (evaporation) of the *B* component from the alloy.

According to Oletta [15], the value describing the potential for evaporation of a liquid component from a metallic alloy is the so-called volatility ratio. In the case of a two-component alloy with an atomic metal vapor system, the volatility ratio is defined by the following equation:

(2) $$\Phi_{B}=\left(\gamma_{B}\frac{p_{B}^{0}}{p_{A}^{0}}\right)\cdot {\left(\frac{M_{A}}{M_{B}}\right)}^{0,5},$$

where Φ_*B*_—volatility ratio of the alloy *B* component; *M_A_*, *M_B_*—atomic (molar) mass of the alloy main component *A* and the evaporating component *B*.

It is generally assumed that the process of ‘i’ component evaporation is possible if the following condition is met:

(3) $$\Phi_{i}>1$$

Metal can evaporate in both atomic and particulate forms. In the latter case, the volatility ratio equation takes the following form:

(4) $$\Phi_{B}={\left(\frac{M_{A}}{M_{B}}\right)}^{0,5}\cdot \left(\frac{p_{B}^{0}}{p_{A}^{0}}\right)\cdot \gamma_{B}\cdot X_{B}^{\left(j-1\right)},$$

where *X_B_*—a molar fraction of the *B* component of the alloy; *j*—the number of atoms in the metal particle *B* in the gaseous phase.

**Figure 3:**
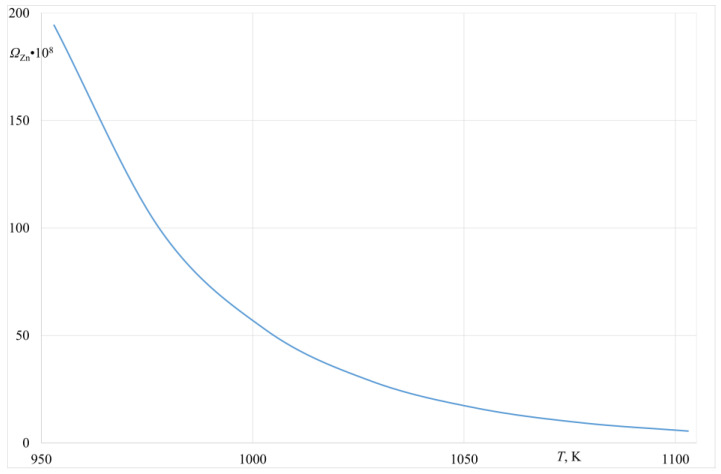
Ω ratio for the Al-Zn alloy (6.3% wt.% Zn).

**Figure 4:**
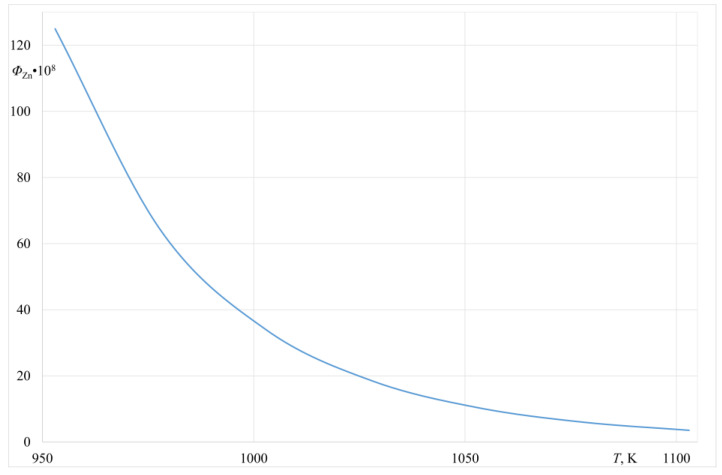
Φ volatility ratio for the Al-Zn alloy (6.3% wt.% Zn).

Figure 3 presents a change of the Ω ratio for the Al-Zn alloy (6.3 wt.% Zn) within the temperature range of 953–1103 K. A similar relation describing the Φ volatility ratio for the same alloy is presented in Figure 4.

The data presented in Figure 3 and Figure 4 indicate that, from the thermodynamics point of view, there is a possibility to evaporate Zinc from the investigated Al-Zn alloy within the assumed temperature range. For the studied alloy, both Ω_*Zn*__/Al_ and Φ_*Zn*_ ratios are >1. The values of the volatility and evaporation ratios presented in Figure 3 and Figure 4 were determined based on the thermodynamics data taken from a study by Plewa [16] and the HSC Chemistry Database [17].

### 2.2. Experimental Procedures

The research experiments were performed on a multi-component Al-Zn5.5MgCu alloy; its composition is presented in Table 1.

The melting furnaces applied in the experiments were the VIM−20 vacuum induction melting furnace and the vacuum furnace with a cold crucible ISM 2–200 (Seco-Warwick, Swiebodzin, Poland).

All the experiments assumed in the research were conducted following a fixed scheme. In the first stage, when the sample was placed in the furnace, the pressure was lowered in the working chamber, and then the charge was heated and melted. Upon reaching the set temperature by the liquid metal, it was maintained in the furnace for 600 s. The temperature measurement was performed using a thermoelectric B-type sensor PtRh30-PtRh6 and an optical pyrometer (PI 1M, Optris, Germany). During the process, a sample of liquid metal was collected at defined intervals. When the experiment finished, the chemical composition of the metal was analysed. For this purpose, the atomic absorption spectrometry method with the ASA solar device was used.

In the VIM unit, the process was performed within the temperature range of 953 K to 1103 K, while the ISM unit experiments were conducted at 953 K. The recorded changes in the furnace operating power ranged from 8 kW to 22 kW and from 70 kW to 130 kW for the VIM furnace and the ISM furnace, respectively. The operating pressure in both units was changed within the range of 10 Pa to 1000 Pa.

## 3. Results

**Figure 5:**
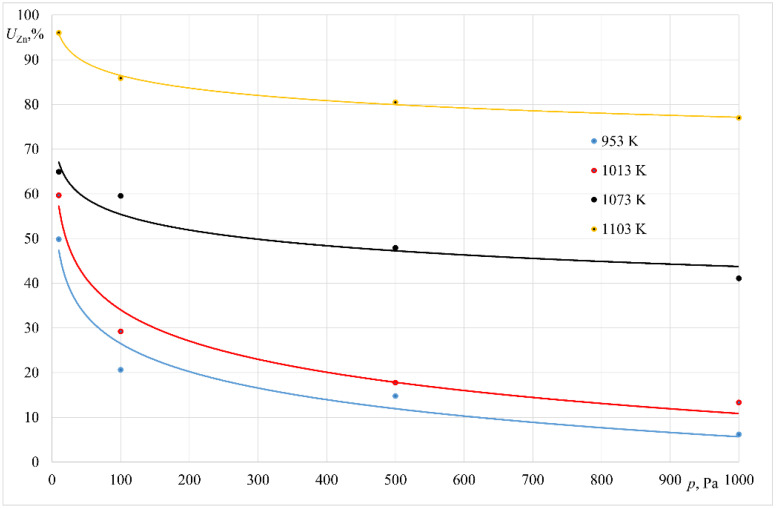
Relative zinc mass loss for the experiments performed in the crucible furnace.

**Figure 6:**
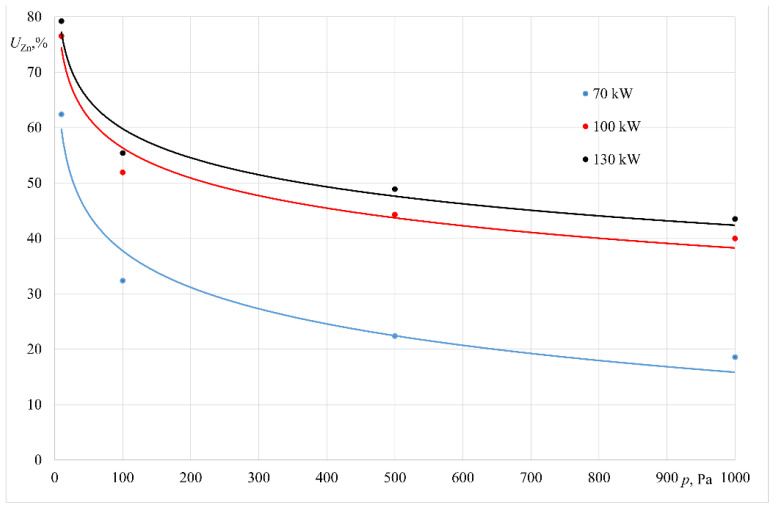
Relative zinc mass loss for the experiments performed in the furnace with a cold crucible.

Results obtained in all experiments are presented in Table 2 and Table 3. In addition to the basic parameters of each experiment, the final Zn content in the alloy and its removal fraction (*U_Zn_*) are shown. Graphic interpretations of these results are shown in Figure 5 and Figure 6.

As indicated by the data presented in Table 2, a raise in the process temperature from 953 K to 1103 K, with a simultaneous reduction in the operating pressure in the vacuum crucible furnace results in an increase in the relative Zinc loss in the alloy from 6.19% to 96.03%.

For the experiments conducted at 935 K, the loss of zinc mass was as follows, depending on the unit applied: 6.19% to 49.84% for the experiments conducted in the crucible furnace; 18.57% to 79.20% for the experiments conducted in the furnace with a cold crucible.

## 4. Discussion

The elimination of impurities in vacuum induction melting is generally observed to follow first-order kinetics [18]. As a result, the overall Zinc elimination rate can be described by the following equation:

(5) $$\frac{dC_{Zn}}{dt}=k_{Zn}\cdot \frac{F}{V}\cdot C_{Zn},$$

This relationship can be written in the following integral form:

(6) $$\int_{0}^{t}\frac{dC_{Zn}}{C_{Zn}}=k_{Zn}\cdot \frac{F}{V}\int_{0}^{t}dt,$$

After integrating Equation (6), we have:

(7) $$2.303\log\frac{C_{Zn}^{t}}{C_{Zn}^{0}}=-k_{Zn}\frac{F}{V}\left(t-t_{0}\right),$$

where *C_Zn_*^0^, *C_Zn_^t^*—zinc concentrations in the alloy, initial and at *t*, respectively (wt.%); *k_Zn_*—overall mass transfer coefficient (ms^−1^); *F*—evaporation surface area (m^2^); *V*—liquid metal volume (m^3^); (*t* − *t*_0_)—duration of the process (s). 

The form of Equation (7) shows that in order to determine the value of the *k_Zn_* coefficient, it is necessary to know the volume of the melt and its surface. In the analysed experiments, within 953 K to 1103 K, the estimated value of the liquid metal volume fell within the range of 420 to 425 cm^3^. The values of liquid aluminium density within the analysed temperature range were determined based on the data in [19,20]. They ranged from 2.37 to 2.41 g cm^−3^.

When analysing the process of zinc evaporation from molten aluminium alloys during their smelting in a vacuum induction furnace, attention should be paid to the issue of increasing the liquid metal surface with the increase in power released in the charge. With greater power, we observe a significant increase in the bath surface due to the formation of a meniscus, which is an effect of the electromagnetic field acting on the liquid metal.

To estimate the size of the surface, the following method of determining the area consisting of four stages was proposed [1]:

- Acquisition of images of the molten metal with the use of a high-speed camera;
- Determination of the resulting meniscus geometry on the basis of the obtained photos;
- Determination of the functions of the curves describing the meniscus for various process parameters;
- Estimation of the surface area using the programs Wolfram Mathematica (F1).

**Figure 7:**
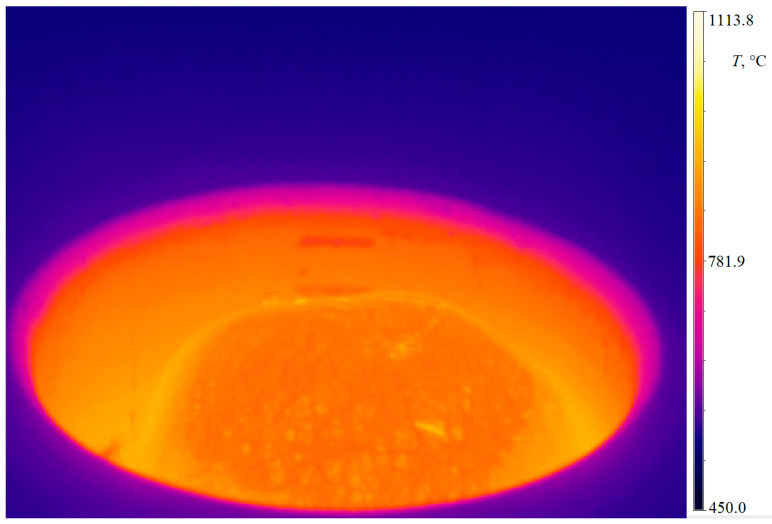
Images of the surface of aluminium melted in the ISM furnace.

As part of the first stage, using a Phantom v5.1 camera and the FLIR thermal imaging camera, a series of photos of the liquid metal was taken during the process for the assumed operating parameters of the furnace. The use of the WebPlotDigitizer 4.1 program allowed us to place points on the photos describing the resulting geometry. Based on this, a line and a function describing the shape of the interface were determined. Figure 7 shows an exemplary photo of liquid aluminium melted in the ISM furnace.

Table 4 summarises the values of the meniscus surface area estimated for all experiments using the proposed calculation method. 

**Figure 8:**
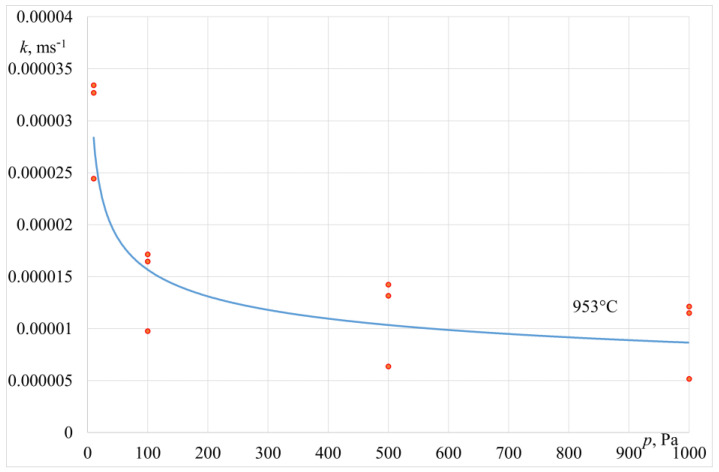
Variation of overall mass transfer coefficient *k_Zn_* vs. pressure (crucible induction furnace).

**Figure 9:**
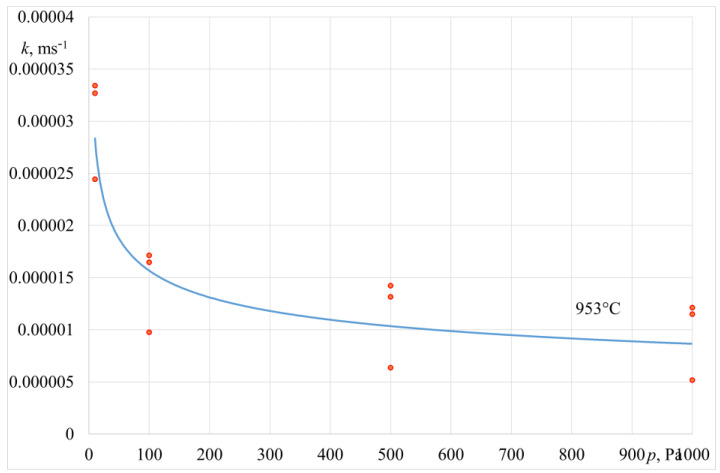
Variation of overall mass transfer coefficient *k_Zn_* vs. pressure (induction furnace with a cold crucible).

The values of the zinc overall mass transfer coefficient *k_Zn_*, determined based on Equation (7), are presented in Table 5 and Table 6. Figure 8 and Figure 9 show a graphic interpretation of changes in the coefficient *k_Zn_* values versus the operating pressure of the crucible induction furnace.

The data in Table 5 and Table 6 show that the reduction in the operating pressure of the furnace working chamber from 1000 Pa to 10 Pa results in an increase in the overall mass transfer coefficient *k_Zn_*. This refers to the experiments carried out in both the crucible induction furnace and the induction furnace with a cold crucible. The values of the *k_Zn_* coefficient obtained in the research are fairly consistent with the literature data reported by other authors studying the process of zinc evaporation from copper, iron, and aluminium alloys melted in the conditions of lower pressure [21,22,23,24]. For the furnace with a cold crucible, the increase in the operating power from 70 kW to 130 kW results in a higher overall mass transfer coefficient *k_Zn_* due to an intensified process of liquid alloy stirring, with a higher furnace power.

### 4.1. Mass Transfer in the Liquid Phase

For a metal which is melted and stirred in the induction furnace, the arsenic mass transfer coefficient *β^l^* can be determined with the use of the recommended Machlin’s equation [25] as follows:

(8) $$\beta^{l}=\left(\frac{8\;D_{Zn}\;\vartheta_{m}}{\pi \;r_{m}}\right),$$

where *ν_m_*—near-surface velocity of inductively stirred liquid metal; *r_m_*—radius of the liquid metal surface (most commonly assumed to be the crucible inner radius); *D_Zn_*—*_Zn_* diffusion coefficient in liquid aluminium.

By analogy to penetration models of mass transfer, Machlin assumed that liquid metal components move along the tangent, i.e., liquid metal–gas or liquid metal–crucible, and the velocity gradient normal to the surface is near the zero value. The equation shows that the *β^l^* value is directly proportional to the near-surface velocity of the liquid metal. Most of the authors who study the evaporation kinetics of inductively stirred metal bath components assumed consistently with Machlin that this velocity is practically independent of the electrical parameters of furnace operation. For induction furnaces with a capacity of up to 1 Mg, the value of *ν_m_* was assumed to be constant and equal to 0.1 ms^−1^. However, further studies showed that this velocity depends on parameters such as the furnace power, current frequency, crucible geometry, or the crucible location in the furnace relative to the inductor [26,27]. In the present paper, the *β^l^* coefficient was determined using the near-surface velocity data from studies by [28,29], in which they were estimated for various metal alloys in experiments carried out in the same melting device [30].

Table 5 and Table 6 present values of the *β^l^* coefficient determined based on Equation (8). They fell within the range of the analysed temperatures and ranged from 3.8 to 5.49 10^−4^ ms^−1^. The value of the zinc diffusion coefficient in liquid aluminium was estimated based on the data contained in the paper [31].

**Figure 10:**
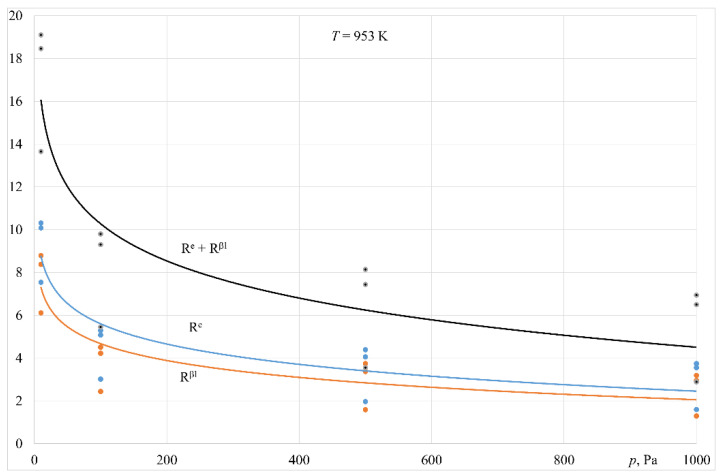
Variation in the resistance of mass transfer in the liquid phase (*R^βl^*) and reaction on the surface of the liquid metal (*R^e^*) in overall resistance of evaporation process vs. pressure (crucible induction furnace).

**Figure 11:**
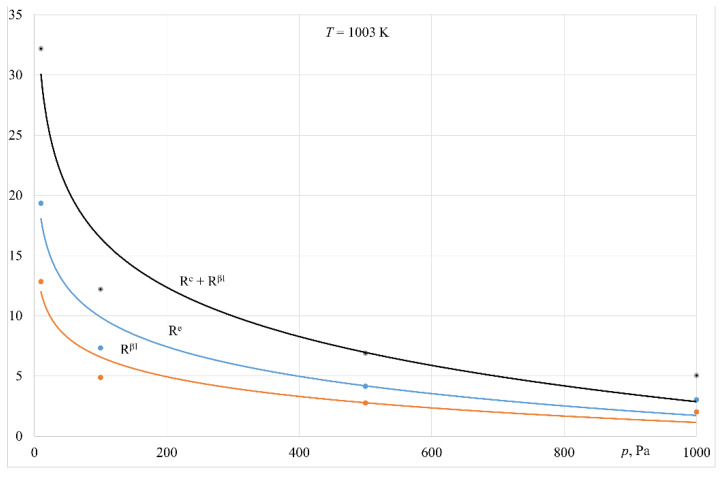
Variation in the resistance of mass transfer in the liquid phase(*R^βl^*) and reaction on the surface of the liquid metal (*R^e^*) in overall resistance of evaporation process vs. pressure (induction furnace with a cold crucible).

Figure 10 and Figure 11 present the estimated transfer resistance in the liquid phase as a fraction of the overall resistance of zinc evaporation for processes carried out at 1003 K and 1103 K. The following equation was applied:

(9) $$R^{l}=\frac{\left(\frac{1}{\beta^{l}}\right)}{\left(\frac{1}{k_{Zn}}\right)}\;\cdot \;100\%,$$

The data in Figure 10 and Figure 11 indicate that for the temperature and pressure ranges in the analysed experiments, zinc evaporation is not determined by mass transfer in the liquid phase. Mass transfer resistance in the liquid metal phase as a fraction of the overall process resistance does not exceed 31%. The pressure decrease is associated with a slightly increased fraction of this resistance. This refers to the experiments conducted in both melting devices.

### 4.2. Evaporation from the Surface

**Figure 12:**
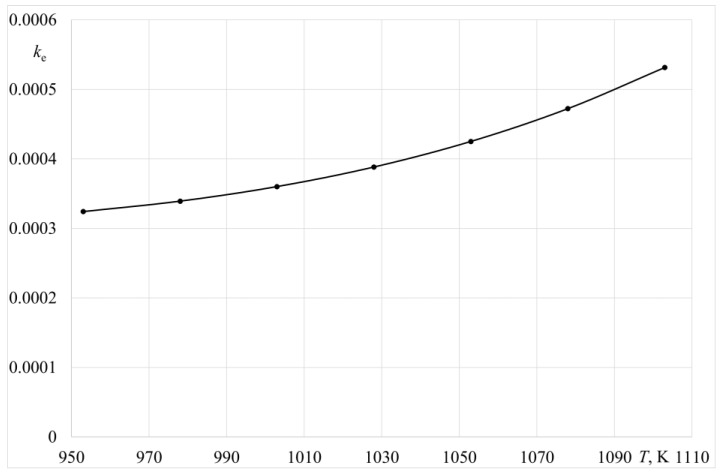
Effects of temperature on the coefficient *k^e^_Zn_* value.

When analysing the rate of zinc evaporation from the liquid alloy surface, the maximum value of rate constant *k^e^_Zn_* was assumed to be described by the following equation:

(10) $$k_{Zn}^{e}=\frac{\alpha \cdot p_{Zn}^{0}\;\gamma_{Zn}\;\cdot \;M_{Al}}{{\left(2\pi RTM_{Zn}\right)}^{0,5}\;\cdot \;\rho_{Al}},$$

where *α*—evaporation constant; *p^0^_Zn_*—equilibrium vapour pressure of zinc over liquid metal; *γ_Zn_*—activity coefficient of zinc in liquid aluminium; *M_Al_*, *M_Zn_*—the molar mass of aluminium and zinc, respectively; *ρ_Al_*—aluminium density. In the present paper, the values of zinc vapour pressures *p*^0^*_Zn_* were determined based on the data from the HSC thermodynamic database [17], and the values of the activity coefficient for zinc in liquid aluminium *γ_Zn_* were determined based on the data reported in a study by [16]. The estimated values of *k^e^_Zn_* are summarised in Table 5 and Table 6 and presented graphically in Figure 12.

Figure 10 and Figure 11 present an example of estimated transfer resistance, associated with a reaction occurring on the surface of the liquid alloy, as a fraction of the overall resistance of the zinc evaporation process for the experiments carried out at 1003 K and 1103 K. The following equation was used:

(11) $$R^{e}=\frac{\left(\frac{1}{k^{e}}\right)}{\left(\frac{1}{k_{Zn}}\right)}\;\cdot \;100\%,$$

Estimation of the resistance *R^e^* as a fraction of the overall process resistance, within the ranges of temperature and pressure applied in this research, showed that it changed from 1.5% to 28% for the experiments performed in the crucible induction furnace, and from 1.5% to 11% for the experiments carried out in the furnace with a cold crucible (only 953 K for this device).

Based on the analysis of the experiments’ findings, it can be concluded that with operating pressures of 100 Pa to 1000 Pa in both melting devices, as well as the analysed temperature range, the process of zinc evaporation from liquid aluminium is determined by mass transfer in the gaseous phase. The overall resistance associated with mass transfer in the liquid phase (*R^βl^*) and with the reaction on the liquid metal surface (*R^e^*) was up to 22% of the overall process resistance (Figure 10 and Figure 11). Pressure lowering to 10 Pa results in an increase in the total resistance (*R^βl^* + *R^e^*) of up to 65% (Figure 10 and Figure 11).

### 4.3. Mass Transfer in the Gaseous Phase

None of the known hydrodynamic models used to describe mass transfer in the gaseous phase enable precise estimation of the mass transfer coefficient *β^g^_Zn_* for the measurement system applied in the research. Similarly, there are no criteria provided in the literature to determine properly this coefficient value.

Therefore, to describe the role of phenomena associated with mass transfer in the gaseous phase in the analysed zinc process, the overpressure ratio (OPR) was used according to the suggestion provided in [32]. This parameter is defined as a ratio of the sum of initial pressures of the bath components over the alloy to the pressure in the furnace working chamber.

(12) $$\mathrm{OPR}=\frac{\sum_{i=1}^{n}p_{i}}{p_{ch}},$$

**Figure 13:**
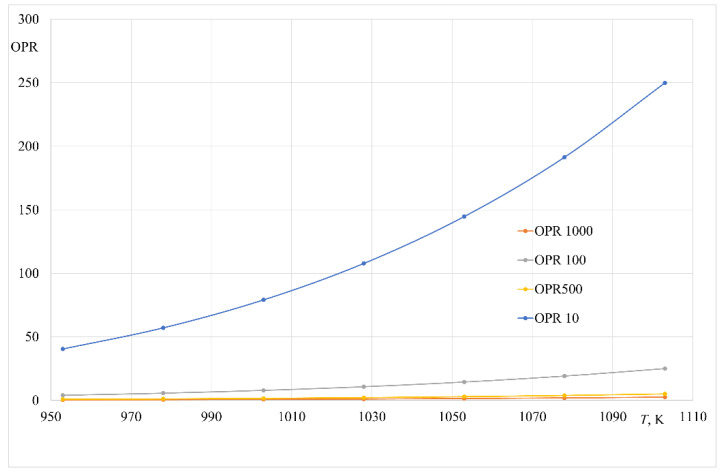
Change in the value of the OPR coefficient for different working pressures of the furnace.

It is assumed that transport in the gaseous phase does not determine the rate of the analysed metal evaporation process under reduced pressure when the following condition is met:

(13) OPR>1,

To estimate the OPR coefficient for the analysed temperature range applied in the experiments, vapour pressures of all volatile components over liquid Al-Zn alloy were considered. The OPR values versus temperature are presented in Figure 13. The data suggest that the analysed process is not determined by mass transfer in the gaseous phase only for the pressure of 10 Pa (OPR > 1).

## 5. Conclusions

As part of the above research, a kinetic analysis of zinc evaporation from aluminium during its smelting in the crucible induction furnace and the induction furnace with a cold crucible was carried out. The analysis involved the following procedures:

- Determination of the values of the overall mass transfer coefficient *k_Zn_* regarding the evaporating component;
- Estimation of the values of mass transfer coefficients in the liquid phase *β^l^* and the evaporation rate constant *k^e^*;
- Determination of the stages defining the investigated process.

The research findings and their analysis led to the following conclusions:

1. For the experiments carried out in the crucible induction furnace, reduction in the operating pressure in the furnace from 1000 Pa to 10 Pa, together with the increase in temperature from 953 K to 1103 K, is accompanied by a decrease in zinc content in the alloy from 6 to 96%, compared with the initial value. Simultaneously, the overall mass transfer coefficient *k_Zn_* increases from 5.15 × 10^−6^ to 1.49 × 10^−4^ ms^−1^.
2. For the experiments carried out in the furnace with a cold crucible (T = 953 K), a reduction in the operating pressure in the furnace from 1000 Pa to 10 Pa resulted in a decrease in zinc content in the alloy from 18 to 80%, compared with the initial value. Simultaneously, the overall mass transfer coefficient *k_Zn_* increases from 5.17 × 10^−6^ to 3.34 × 10^−5^ ms^−1^.
3. For comparison, the experiments carried out in the crucible induction furnace at 953 K showed a reduction in zinc content in the alloy from 6 to 50%, which means that more intense zinc evaporation is seen in the furnace with a cold crucible. This effect is caused by a far larger evaporation surface (liquid metal surface) for this device (Table 4).
4. The rate of analysed zinc evaporation within 100 Pa to1000 Pa is controlled by the mass transfer rate in the gaseous phase. The overall resistance associated with mass transfer in the liquid phase (*R^βl^*) and with the reaction on the liquid metal surface (*R^e^*) was up to 22% of the overall process resistance for this pressure range.
5. For 10 Pa, the overall resistance associated with mass transfer in the liquid phase (*R^βl^*) and with the reaction on the liquid metal surface (*R^e^*) constitutes nearly 70% of the overall process resistance.

## Figures and Tables

**Table 1:** Chemical composition of the investigated alloy.

Content of Basic Alloy Components. wt.%									
Zn	Mg	Cu	Mn	Fe	Si	Cr	Ti	Zr	Al
6.3	2.5	1.6	0.3	<0.5	<0.4	0.23	<0.05	0.01	residue

**Table 2:** Results of the experiments performed in the crucible induction furnace (VIM).

No.	*T*, K	*p*, Pa	Final Zinc Concentration, wt.%	*U_Zn_*, %
1	953	1000	5.91	6.19
2	953	500	5.37	14.76
3	953	100	5.00	20.63
4	953	10	3.16	49.84
5	1013	1000	5.46	13.33
6	1013	500	5.18	17.77
7	1013	100	4.46	29.20
8	1013	10	2.54	59.68
9	1073	1000	3.71	41.11
10	1073	500	3.28	47.93
11	1073	100	2.55	59.52
12	1073	10	2.21	64.92
13	1103	1000	1.45	76.98
14	1103	500	1.23	80.47
15	1103	100	0.89	85.87
16	1103	10	0.25	96.03

**Table 3:** Results of the experiments performed in the induction furnace with a cold crucible.

No.	*T*, K	*p*, Pa	Operating Power, kW	Final Zinc Concentration, wt.%	*U_Zn_*, %
1	953	1000	70	5.13	18.57
2	953	500	70	4.89	22.38
3	953	100	70	4.26	32.38
4	953	10	70	2.37	62.38
5	953	1000	100	3.78	40.01
6	953	500	100	3.51	44.28
7	953	100	100	3.03	51.90
8	953	10	100	1.48	76.50
9	953	1000	130	3.56	43.49
10	953	500	130	3.22	48.88
11	953	100	130	2.81	55.39
12	953	10	130	1.31	79.20

**Table 4:** Values of the surface area of meniscus formed for Al-Zn alloys, estimated with the use of the proposed calculation method.

No.	*p*, kW	Furnace	F, cm^2^
1	8	VIM	88.1
2	12	VIM	92.4
3	17	VIM	108.7
4	22	VIM	155.0
5	70	ISM	280.0
6	100	ISM	311.8
7	130	ISM	330.3
8	8	VIM	88.1
12	12	VIM	92.4

**Table 5:** The overall mass transfer coefficient *k_Zn_* as well as the coefficients *β^l^_Zn_* and *k*^e^*_Zn_* (crucible induction furnace).

*T*, K	*p*, Pa	*k_Zn_*, m s^−1^	*β^l^_Zn_*, m s^−1^	*k*^e^*_Zn_*, m s^−1^
5953	1000	0.00000515	0.000549	0.000324
953	500	0.0000129	0.000549	0.000324
953	100	0.0000185	0.000549	0.000324
953	10	0.0000556	0.000549	0.000324
1013	1000	0.0000109	0.000542	0.00036
1013	500	0.0000149	0.000542	0.00036
1013	100	0.0000264	0.000542	0.00036
1013	10	0.0000696	0.000542	0.00036
1073	1000	0.0000351	0.00052	0.000425
1073	500	0.0000426	0.00052	0.000425
1073	100	0.0000592	0.00052	0.000425
1073	10	0.0000685	0.00052	0.000425
1103	1000	0.0000673	0.000478	0.000531
1103	500	0.0000748	0.000478	0.000531
1103	100	0.0000896	0.000478	0.000531
1103	10	0.000149	0.000478	0.000531

**Table 6:** The overall mass transfer coefficient *k_Zn_* and the coefficients *β^l^_Zn_* and *k*^e^*_Zn_* (induction furnace with a cold crucible).

*T*, K	*p*, Pa	*p*, kW	*k_Zn_*, m s^−1^	*β^l^_Zn_*, m s^−1^	*k*^e^*_Zn_*, m s^−1^
953	1000	70	0.00000517	0.0004	0.000324
953	500	70	0.00000636	0.0004	0.000324
953	100	70	0.000009768	0.0004	0.000324
953	10	70	0.00002444	0.0004	0.000324
953	1000	100	0.00001151	0.00039	0.000324
953	500	100	0.00001316	0.00039	0.000324
953	100	100	0.00001646	0.00039	0.000324
953	10	100	0.00003268	0.00039	0.000324
953	1000	130	0.00001213	0.00038	0.000324
953	500	130	0.00001423	0.00038	0.000324
953	100	130	0.00001714	0.00038	0.000324
953	10	130	0.0000334	0.00038	0.000324

## Data Availability

Data sharing is not applicable to this article.
